# Antibody-Mediated Targeting of Antigens to Intestinal Aminopeptidase N Elicits Gut IgA Responses in Pigs

**DOI:** 10.3389/fimmu.2021.753371

**Published:** 2021-10-14

**Authors:** Hans Van der Weken, Raquel Sanz Garcia, Niek N. Sanders, Eric Cox, Bert Devriendt

**Affiliations:** ^1^ Laboratory of Immunology, Faculty of Veterinary Medicine, Ghent University, Ghent, Belgium; ^2^ Laboratory of Gene therapy, Faculty of Veterinary Medicine, Ghent University, Ghent, Belgium

**Keywords:** oral vaccination, aminopeptidase N, epithelial targeting, ETEC, recombinant antibody, mucosal immunity

## Abstract

Many pathogens enter the host *via* the gut, causing disease in animals and humans. A robust intestinal immune response is necessary to protect the host from these gut pathogens. Despite being best suited for eliciting intestinal immunity, oral vaccination remains a challenge due to the gastrointestinal environment, a poor uptake of vaccine antigens by the intestinal epithelium and the tolerogenic environment pervading the gut. To improve uptake, efforts have focused on targeting antigens towards the gut mucosa. An interesting target is aminopeptidase N (APN), a conserved membrane protein present on small intestinal epithelial cells shown to mediate epithelial transcytosis. Here, we aimed to further optimize this oral vaccination strategy in a large animal model. Porcine APN-specific monoclonal antibodies were generated and the most promising candidate in terms of epithelial transcytosis was selected to generate antibody fusion constructs, comprising a murine IgG1 or porcine IgA backbone and a low immunogenic antigen: the F18-fimbriated *E. coli* tip adhesin FedF. Upon oral delivery of these recombinant antibodies in piglets, both mucosal and systemic immune responses were elicited. The presence of the FedF antigen however appeared to reduce these immune responses. Further analysis showed that F18 fimbriae were able to disrupt the antigen presenting capacity of intestinal antigen presenting cells, implying potential tolerogenic effects of FedF. Altogether, these findings show that targeted delivery of molecules to epithelial aminopeptidase N results in their transcytosis and delivery to the gut immune systems. The results provide a solid foundation for the development of oral subunit vaccines to protect against gut pathogens.

## Introduction

Most pathogens invade the host at the mucosal surfaces, such as the gut. Frontline protection against these enteropathogens requires robust intestinal immune responses at the site of infection, more specific pathogen-specific secretory immunoglobulin A (SIgA) (1). In contrast to systemic administration, delivery of vaccines to the intestinal mucosa can elicit protective SIgA responses at both local and distal mucosal sites as well as systemic immunity (1–3). Oral vaccines have many advantages: they avoid the use of needles, which reduces the need for trained personnel and the risk of transmitting blood borne diseases. They also increase patient compliance and often do not require refrigerated storage, resulting in easier transport and delivery to remote places (2, 4). Current oral vaccines consist of either inactivated or live-attenuated organisms which pose several risks, such as severe inflammatory reactions, uncontrolled replication, the possibility of reversion to virulence or the risk of infection in immunocompromised patients. Thus, the development of new vaccination strategies has shifted to the use of safer subunit vaccines. Nevertheless, oral vaccination and the induction of robust protective immune responses faces many hurdles. Vaccine antigens not only need to survive the gastric pH and degradation by proteolytic enzymes in the gastrointestinal tract, they also must reach the gut-associated lymphoid tissue. However, the small intestinal epithelial barrier restricts uptake of macromolecules, leading to a poor uptake of vaccines at the intestinal surfaces. In addition, without proper activation and correct dosing, tolerance is induced rather than protective immunity (1). To overcome these challenges in oral vaccination, current efforts are focused on different encapsulation strategies to preserve antigen stability in the gut, novel mucosal adjuvants to surmount tolerance or targeting antigens to intestinal cell populations to enhance vaccine uptake (5). For instance, the glycoprotein-2 (GP2) protein is specifically expressed on the apical side of mature M cells and can recognize the bacterial FimH, a component of type I pili on the bacterial outer membrane. Uptake of FimH+ bacteria by M-cells *via* GP2 was able to initiate mucosal immune responses in mice (6). An alternative strategy would be to target vaccine antigens towards enterocytes, since these cells are more abundant than M cells in the small intestinal epithelium (1, 7). For example, targeting receptors involved in transcytosis such as the neonatal Fc-receptor (FcRn) enabled the uptake of antigen-bound IgG Fc-fragments (8, 9). Another interesting target is aminopeptidase N (APN; CD13). In enterocytes, this membrane glycoprotein is involved in digestive processes by removing N-terminal amino acids from peptides (10). APN is also expressed on specific subsets of dendritic cells in humans, pigs and mice, which play a central role in the induction of adaptive immune responses (11–13). Our previous research identified APN as a receptor for F4 fimbriae and was shown to be involved in the epithelial transcytosis of these fimbriae. Interestingly, oral administration of purified F4 fimbriae to piglets triggered protective SIgA responses (14). Moreover, delivery of antigens and microparticles to aminopeptidase N by different antibody formats facilitated their uptake by the small intestinal epithelium and elicited strong immune responses in piglets upon oral administration (15–18).

Here, we aimed to further optimize this oral vaccine strategy by specifically targeting a clinically relevant antigen towards APN using monoclonal antibody constructs. To this end, we generated several APN-specific monoclonal antibodies and characterized their interaction with APN. From these monoclonal antibodies (mAbs), we selected the best performing candidate and generated different fusion constructs with a mouse IgG1 or pig IgA backbone. These constructs were genetically linked with the FedF tip adhesin from F18 fimbriated *E. co*li, which is a clinically relevant but low immunogenic antigen and evaluated their ability to trigger immune responses in piglets upon oral administration (17, 19).

## Materials and Methods

### Generation of Monoclonal Antibodies

Immunizations with porcine kidney APN (Sigma) and hybridoma generation were carried out by Monash University. Mother clones were subcloned and 6 different clones were selected and further expanded. Secreted antibodies were subsequently purified from the culture supernatant by protein G affinity chromatography (GE healthcare). Monoclonal antibody isotypes were determined using the mouse IgG isotyping ELISA kit (Iso-2, Sigma).

A vector coding for the α-APN-mIgG1-FedF fusion antibody was generated by Genscript. Briefly, the heavy chain of an APN-specific mouse monoclonal antibody (clone IMM013) was fused to the tip adhesin FedF_15-165_ of F18 fimbriae (PDB entry: 4B4P) using a (G_4_S)_3_-flexible linker and cloned into MCS2 of the pVITRO1-neo-mcs vector using CloneEZ^®^ seamless cloning technology. Then, the light chain of the same clone (IMM013) was cloned into MCS1 of the same vector to get the final α-APN-mIgG1-FedF expression vector. After stable transfection into CHO cells, the best producing clones were selected by serial dilution and further expanded. Secreted antibodies were purified from cell culture supernatant using protein A affinity chromatography (GE Healthcare).

The chimeric α-APN-pIgA-FedF and pIgA-FedF control construct were generated as described previously, using the variable regions of the IMM013 clone and the porcine constant light (AAA03520.1) and porcine IgA heavy (AAA65943.1) chains (20). The pIgA-FedF control construct was derived from the IMM013 clone, but contained a single mutation (G100D; MUT7) in the CDR3H region, resulting in loss of binding towards APN (**Figures S1B, C**). Secreted antibody was purified using ammonium sulphate precipitation between 40 and 46% saturation and dialyzed against PBS.

### Affinity Measurements

Affinity measurements were performed using bio-layer interferometry (BLI; Octet RED96). Here, 10 µg/ml of the ligand (biotinylated porcine APN; 1:3 ratio) was first bound on a high precision streptavidin (SAX) biosensor soaked in PBS, followed by the addition of the analyte (mAbs) at 100 nM in PBS + 0.2% Tween-20 + 1% BSA (PBST+BSA). Analyzed data was fitted with a 1:1 local full fit.

### APN-Specific Binding Assays

Binding of mAbs towards purified APN was performed with ELISA as described (18). Binding of mAbs towards membrane-bound APN on BHK-APN cells was analyzed by flow cytometry (Cytoflex, Beckman Coulter) as described (18), with slight modifications. Cells were incubated with mAbs (10 µg/ml) and detected with a fluorescein isothiocyanate (FITC)-conjugated sheep α-mouse IgG (whole molecule) F(ab’)2 fragment (1:100 dilution) (Merck, F2883). Isotype control mouse IgG1 and IgG2a antibodies (in-house) were used as controls.

### Porcine Small Intestinal Explants

Tissue explants from porcine ileum were obtained as described (17). Antibodies (40 µg) were added to the explants for 30 minutes at 37°C and 5% CO_2_. Upon this incubation period, the explants were washed with PBS, placed in methocel, snap frozen in liquid nitrogen and stored at -80°C until use.

### Gut Ligated Loop Experiments

In total, six female, 5-week-old piglets were used to assess the uptake of α-APN-mIgG1 (clone IMM013) in gut ligated loops as described (21). Three of these animals were used in a preliminary study to locate the mesenteric lymph nodes draining each area of the gut and to study the kinetics of the uptake in the gut ligated loops after different incubation times. Briefly, following anesthesia and laparotomy, the jejunum was localized and three 3 cm loops with 20 cm intervals between each loop were made avoiding Peyer’s patches. Blood supply was assured by placing the ligatures between the mesenteric arcades. For the location of the draining MLN, 5% Evans Blue was injected subserosally between the ligatures of each loop of the small intestine. One milligram of fluorescently labelled (DyLight TM 755, Thermo Fisher Scientific) α-APN mAb (clone IMM013) or an IgG1 isotype control (in house; clone 19C9) (22) were diluted in 3 ml PBS and injected in the lumen of the loops. A loop injected with 3 ml PBS was used as a negative control. Upon injection, each loop was returned to the abdominal cavity and the abdomen was closed. After a 5h incubation, the animals were euthanized with an overdose of sodium pentobarbital 20% (60 mg/2.5kg; Kela) and tissue samples were collected. Loops and draining MLN were imaged using an IVIS Lumina II fluorescent imaging system. Tissues were kept on ice protected from light until imaging. Following, tissue samples were embedded in 2% Methocel^®^ MC (Fluka), snap frozen in liquid nitrogen and stored at -80°C until use.

### Immunohistochemistry

For the endocytosis experiments using the BHK-APN cell line, cells (1.0 x 10^5^ cells/well in 1 ml culture medium) were seeded in 24-well plates on top of a sterile cover slip and incubated until a monolayer was formed. Cells were washed twice with ice-cold PBS and stored on ice before the α-APN-mIgG1 (40 µg/ml) was added. After 60 min incubation at 4°C, cells were washed 3 times with ice-cold PBS + 1% FCS and incubated for 30 min at 37°C, 5% CO_2_ in warm culture medium. Before or after incubation at 37°C, cells were washed twice with ice-cold PBS and fixated for 10 min with 500 µl 4% paraformaldehyde. Next, presence of the antibody on the cell membrane was detected with an AF568-conjugated α-mouse IgG(H+L) (2 µg/ml; Invitrogen, A-11004) for 30 min at room temperature (RT). After three washes with PBS + 1% FCS, cells were permeabilized with 250 µl 0.2% Triton-X100 for 2 min and washed 3 times with PBS + 1% FCS. Intracellular α-APN-mIgG1 was then detected using a FITC-conjugated sheep α-mouse IgG F(ab’)2 fragment (1:100 dilution; Merck, F2883) for 1h at RT. The nucleus was counterstained with Hoechst (10 µg/ml) for 2 min. After three washes, the cover slip was mounted on a microscope slide in mounting solution (Dabco).

For staining of tissue sections, cryosections (10 µm) were cut with a cryotome (Leica CM3050 S), placed on APES-coated glass slides and fixated in aceton for 10 minutes at -20°C. Tissue sections were then washed with 50 mM ammonium chloride (pH 8.0) for 30 min followed by a short PBS wash. Next, tissue sections were blocked with PBS + 10% sheep serum or goat serum for 30 minutes in a humid cell at 37°C. To assess binding of the different mAbs, sections were incubated for 1h at 37°C with these mAbs (10 µg/ml). After incubation, a secondary FITC-conjugated sheep α-mouse IgG F(ab’)2 fragment (1:100 dilution; Merck, F2883) was added for 1h at 37°C. For staining and the α-APN-mIgG1 uptake experiment with explant tissue, a rabbit pAb to wide-spectrum cytokeratin (1:100 dilution; Abcam, ab9377) was added for 1h at 37°C, followed by a secondary FITC-conjugated sheep α-mouse IgG F(ab’)2 fragment (1:100 dilution; Merck, F2883) and a Texas Red-conjugated goat α-rabbit IgG(H+L) (1:100 dilution, Invitrogen). To stain immune cells, mAbs to MHC-II (clone MSA3, IgG2a, 15 ug/ml, in house), CD11R1 (biotinylated, clone MIL4, IgG1, 15 ug/ml, Bio-Rad) and CD172a (biotinylated, clone 74-22-15a, IgG1, 10 ug/ml, in house) were added and incubated for 1h at 37°C, followed by another incubation for 1 h at 37°C with FITC-conjugated sheep α-mouse IgG2a (Invitrogen, Catalog #31634, 1/100 dilution) or streptavidin-Texas Red (Invitrogen, S872, 1/50 dilution).

Slides were washed with PBS between each step, counterstained with Hoechst (10 µg/ml) for 2 min and mounted on a microscope slide in mounting solution (Dabco). Images of explants were taken with a confocal microscope (Leica). Other images were taken with a fluorescent microscope (Leica). Images were analyzed and processed using Fiji.

### Animals and Immunization Procedures

Twenty-five conventionally reared piglets (Belgian Landrace x Pietrain) from a Belgian farm were weaned at 3 weeks and transported to our facilities. These animals were screened to be mouse IgG1, cholera toxin and F18 fimbriae seronegative. Piglets receiving the FedF constructs were also screened to be F18 receptor positive using FUT1 genotyping (23). The piglets were housed in isolation units and treated with colistin (Colivet quick pump ^®^, 6,4mg/kg bodyweight) for 5 days before the start of the experiment. Animals were randomly divided in five groups of 5 animals: 1) a mouse IgG1 (mIgG1) isotype control mAb (clone 19C9), 2) an APN-targeted mouse IgG1 mAb (α-APN-mIgG1), 3) an α-APN-mIgG1-FedF fusion construct and 4) the α-APN-pIgA-FedF or 5) pIgA-FedF chimeric mouse-porcine IgA fusion constructs. The piglets were orally immunized on three consecutive days followed by a booster immunization 14 days post primary immunization (dppi). All immunizations were adjuvanted with 50 µg cholera toxin (Merck, C8052). The gastric pH was neutralized by administration of Omeprazole (20 mg) 24 hours before each immunization and animals were deprived of feed and water 3 hours before the immunizations. Animals were immunized by oral administration with a syringe with 1 mg mIgG1 isotype control or α-APN-mIgG1 and 1.2 mg α-APN-mIgG1-FedF, α-APN-pIgA-FedF or pIgA-FedF in 10 ml PBS to account for equimolar ratios. Blood was collected at 0, 9, 14, 21 and 28 dppi to analyze serum antibody responses by ELISA and assess the presence of antigen-specific IgA+ antibody secreting cells (ASC) in the peripheral blood mononuclear cell (PBMC) population. At 28 dppi animals were euthanized by intravenous injection of sodium pentobarbital 20% (60mg/2.5kg; Kela) and upon exsanguination intestinal tissues were collected.

For the isolation of intestinal antigen presenting cells, 3 to 4 conventionally reared piglets (Belgian Landrace x Pietrain) from a Belgian farm were euthanized by intravenous injection of sodium pentobarbital 20% (60mg/2.5kg; Kela) and upon exsanguination small intestinal jejunal tissue was collected.

### Antigen-Specific Serum Antibody Responses

Blood was taken from the jugular vein into a gel and clot activator tube (Vacutest, Kima). After 1h incubation at RT, tubes were centrifuged and serum was collected, inactivated at 56°C for 30 minutes and kaolin treated. Serum samples were stored at -20°C until use. Maxisorp microtiter plates (96-well, Life Technologies) were coated with mouse IgG1 monoclonal antibody (19C9 or IMM013, 6 µg/ml) or FedF (in house, 5 µg/ml) in PBS for 2h at 37°C. FedF was purified as described previously (17). Upon overnight blocking at 4°C in PBS supplemented with 0.2% Tween80 and 3% BSA, the serially diluted serum samples were added in dilution buffer (PBS + 0.2% Tween20 + 3% BSA) to the wells. Upon incubation for 1 h at 37°C, plates were washed and incubated for 1 h at 37°C with HRP-conjugated mouse α-pig IgG (1/1000; MabTech; Nacka Strand, Sweden) or IgA (1/10000; Bethyl; Montgomery, Texas, U.S). Following 3 washes, ABTS was added and the optical density was measured at 405 nm after 60 min incubation at 37°C using a spectrophotometer (Tecan SpectraFluor). Serum was serially diluted starting at 1/30 for IgG1 and IMM013 responses and 1/10 for FedF serum responses. Titer values were obtained by calculating the non-linear regression curve and using a cut off value 0.2.

### Antigen-Specific Antibody Secreting Cells in the Intestinal Tissues

Mononuclear cells (MCs) were isolated from blood (PBMC), mesenteric lymph nodes (MLN), jejunal Peyer’s Patches (JPP), jejunal lamina propria (JLP), ileal Peyer’s Patches (IPP) and ileal lamina propria (ILP) and processed as described (24, 25). The obtained cell suspensions were filtered through a 70 µm cell strainer and the MCs were isolated by density gradient centrifugation on Lymphoprep (Alere Technologies, Oslo, Norway) for 25 minutes at 800g and 18°C. Isolated MCs were resuspended at 2.5x10^6^ cells/ml (PBMC and MLN) or 5x10^6^ cells/ml (other tissues) in CTL-Test™ B-medium (Cellular Technology Limited, Cleveland, USA). MultiScreen filter plates (96-well format, MAIPA4510, Millipore) were activated with 70% ethanol for 30 seconds, washed twice with ultrapure (UP) water and coated overnight at 4°C with 10 µg/ml mouse IgG1 (in house) or 10 µg/ml FedF. Upon washing, the plates were incubated for 2h at 37°C with CTL-test B medium. Mononuclear cells (5x10^5^ cells/well) from each tissue were added to the wells and incubated for 18h at 37°C, 5% CO2 in a humidified atmosphere. Cells were then removed by intensive washing with PBS containing 0.1% Tween20. Upon washing, HRP-conjugated α-pig IgG (1/1000; MabTech) or IgA (1/10000; Bethyl) was added in assay buffer (PBS containing 0.1% Tween20 and 0.1% BSA) and incubated for 1 hour at RT. Finally, 3,3′,5,5′-Tetramethylbenzidine (TMB) substrate for membranes (Sigma) was added to the wells after three washes. The reaction was stopped by intensive washing with UP water and the plates were allowed to dry overnight at 4°C. Images were taken using an immunospot reader (Luminoskan) and spots were counted manually.

### ETEC Virulence Factors

F4 and F18 fimbriae were purified from the F4^+^ ETEC strain IMM01 (0147:F4ac^+^, LT^+^STa^+^STb^+^) and the F18^+^ VTEC reference strain F107/86 (O139:K12:H1, F18ab^+^, SLT^-^IIv^+^), respectively, as previously described (24, 26). Briefly, bacteria were grown in tryptone soya broth (TSB; Oxoid Hampshire, UK) for 18h at 37°C and 85 rpm. Subsequently, the fimbriae were isolated from the bacteria by mechanical shearing. After ammonium sulphate precipitation, the fimbrial proteins were dialysed, filtrated and stored at -20°C. The protein concentration of the purified ETEC virulence factors was determined with a BCA assay and the purity was assessed by SDS-PAGE and coomassie staining.

### Isolation of Intestinal Antigen-Presenting Cells

Monomorphonuclear cells (MCs) were isolated from the jejunal lamina propria (LP) as described above. APCs were further enriched from the MC fraction by immunomagnetic cell separation (MACS; Miltenyi Biotec, Bergisch Gladbach, Germany). LPMCs were labelled with an anti-MHCII mAb (clone MSA3, IgG2a) and goat anti-mouse IgG microbeads (Miltenyi Biotec). MHCII^+^ cells were retained within a LS column (Miltenyi Biotec) placed in a magnetic field. After washing, the cells were flushed out and stained with anti-SIRPα-DyLight649 (clone 74-12-15, IgG1; DyLight649 conjugation kit, ThermoScientific) and anti-CD16-FITC (IgG1; AbD Serotec, UK). SytoxBlue (1 mM; Invitrogen) was used to stain dead cells according to the manufacturer’s instructions. MHCII^+^SIRPα^+^CD16^hi^ (CD16^hi^) and MHCII^+^SIRPα^+^CD16^+^ (CD16^+^) LPMCs were FACS purified (FACS AriaIII; BD Biosciences, Erembodegem, Belgium). Post-sort analysis revealed a >95% purity of both populations. Sorted cells were stained with anti-human CD68 mAb (IgG2b; eBioscience, Y1/82A) and anti-mouse IgG_2b_-AF594 (Invitrogen, A21145). Nuclei were counterstained with Hoechst (10 µg/ml). Cells were imaged with a fluorescent microscope.

### T-Cell Presentation Assay

The enriched CD16^hi^ and CD16^+^ LP APCs were cultured in round-bottomed 96-well plates at 1.0 x 10^4^ cells/well in DMEM (Gibco) supplemented with 10% FCS, 1% P/S and 20 µg/ml gentamycin. The cells were stimulated for 24h with 1 μg purified F4 fimbriae or F18 fimbriae at 37°C in a humidified atmosphere at 5% CO_2_. Stimulated CD16^hi^ and CD16^+^ cells were washed and subsequently cocultured with CD6^+^ T-cells to analyze their allogeneic T-cell stimulatory capacity as previously described (27). Briefly, PBMCs were purified from heparinized blood samples from an unrelated pig by lymphoprep density gradient centrifugation. CD6^+^ T-lymphocytes were further enriched from the PBMC cell fraction with immunomagnetic cell sorting (MACS system) and anti-CD6 mAbs (28). Next, 1.0 x 10^5^ CD6^+^ T-cells were added to the stimulated intestinal APC populations in DMEM supplemented with 10% FCS, P/S and 20 µg/ml gentamycin (proliferation medium). APCs and CD6^+^ T-cells alone were used as a control for background proliferation, while ConA-stimulated T-cells (5 µg/ml, Sigma) were used a positive control. After 5 days, the cocultures were pulse-labeled with 1 µCi/well [^3^H]methyl-thymidine (Amersham ICN, Bucks, UK) for another 18h. Cells were harvested onto glass fiber filters (Perkin-Elmer, Life Science, Brussels, Belgium) and the [^3^H]methyl-thymidine incorporation was measured using a β-scintillation counter (Perkin-Elmer). The stimulation index was calculated by dividing the mean counts per minute (cpm) of the stimulated conditions by the mean cpm of mock-stimulated iAPCs-CD6+ T-cell co-cultures.

### Data Analysis

The data were analyzed using GraphPad Prism software version 7. Differences in the frequency of ASCs between different groups were analyzed using the Kruskal-Wallis test. Serum responses between groups and between days were analyzed using a Two-way ANOVA with repeated measures. Differences in T-cell proliferation were assessed *via* One-way ANOVA, with LSD *post-hoc* analysis. Homogeneity of variances was assessed with Levene’s test. Multiple comparisons were corrected using the Two-stage linear step-up procedure of Benjamini, Krieger and Yekutieli. Differences were considered significant when the adjusted p-value <.05.

## Results

### Characterization of APN-Specific Monoclonal Antibodies

**Figure 1:**
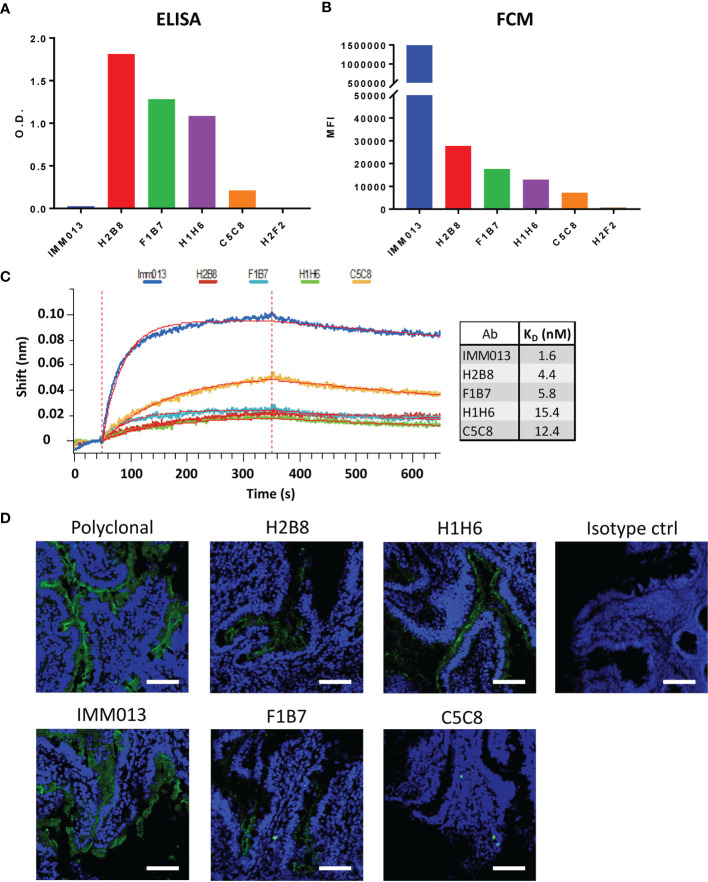
Binding profiles of APN-specific monoclonal antibodies to aminopeptidase N. Binding to porcine aminopeptidase N (APN) was analyzed using **(A)** enzyme-linked immunosorbent assay (ELISA) with purified kidney APN and **(B)** flow cytometry using an APN-expressing cell line. O.D.: Optical density; MFI: Mean fluorescence intensity. O.D. values are subtracted from mean background absorbance. MFI values are subtracted from relevant isotype controls. **(C)** Binding kinetics of several mAbs using bio-layer interferometry (BLI) with resulting affinity (KD) values. Shift in wavelength (nm) is given over time (s). **(D)** Jejunal cryosections, stained with different antibodies and detected with a FITC-conjugated anti-mouse or anti-rabbit IgG (green). Mouse IgG1 and IgG2a antibodies were used as isotype controls. Nuclei were counterstained with Hoechst (blue). Data is representative for 3 animals. Scale bar = 100 µm.

Using standard techniques, 6 hybridoma clones were obtained and further characterized. Although these clones recognized APN in an initial screening, upon their purification clone H2F2 failed to recognize porcine APN in ELISA. Clone H2B8 showed the strongest binding, while IMM013 showed the weakest binding, with optical density (O.D.) values barely above the detection limit (**Figure 1A**). Next, flow cytometry analysis was performed using an APN-expressing cell line (BHK-APN). Surprisingly, all monoclonal antibodies showed a similar binding profile as compared to ELISA, except for IMM013 (**Figure 1B**). While the latter was barely detectable in ELISA, it showed the best binding to membrane bound APN, indicating that purified kidney APN might differ from membrane-bound APN in epitope accessibility. As clone H2F2 also did not bind to BHK-APN cells, it was excluded from further analyses. The affinity of the remaining clones was determined using bio-layer interferometry (BLI) (**Figure 1C**). These results were similar to flow cytometry with IMM013 having the strongest affinity (K_D_) value in the low nanomolar range (**Figure 1D**).

As these monoclonal antibodies might be used for the delivery of vaccine antigens to the small intestinal epithelium, we assessed their ability to recognize APN on small intestinal jejunum and ileum. IMM013 showed the best binding to APN present on the apical side of the small intestinal enterocytes. H2B8, F1B7 and H1H6 showed an intermediate binding, while C5C8 showed a very weak binding (**Figure 1D** and **Supplementary Figure S2**). These binding profiles were very similar to our flow cytometry data, further confirming the importance of using membrane-bound APN to assess the binding capacity of APN-specific mAbs.

### *In Vitro* and *In Vivo* Behavior of APN-Targeted mAb

**Figure 2:**
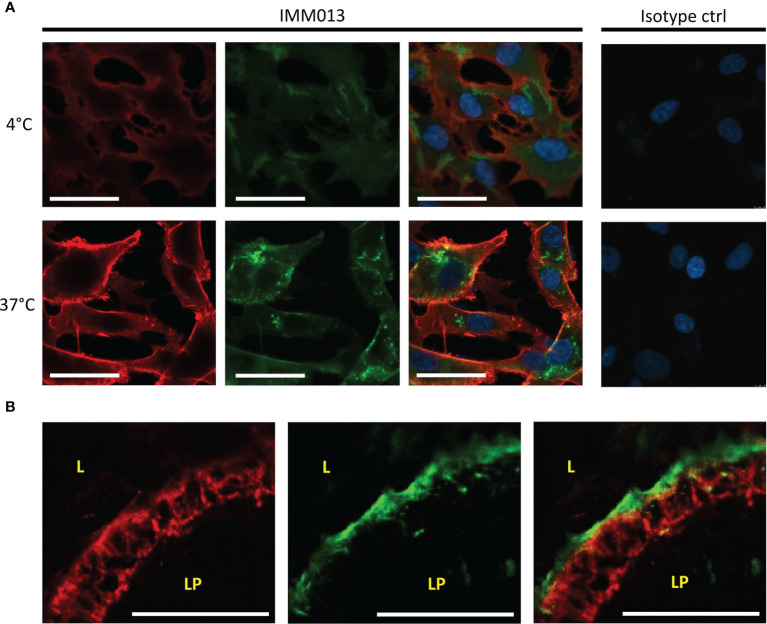
*In vitro* and *ex vivo* uptake of APN-targeted mAb. Fluorescence microscopy images of **(A)** an APN-expressing cell line (BHK-APN) after binding of IMM013 or isotype control at 4°C (top) and after incubation at 37°C (bottom) for 30 minutes. Antibodies were detected with an AF561- conjugated anti-mouse IgG before (red) and with a FITC-conjugated anti-mouse IgG after (green) permeabilization of the cell membrane. Nuclei were counterstained with Hoechst (blue). **(B)** Confocal images of Ileal explants after 30 minutes incubation with IMM013 at 37°C, detected with a FITC-conjugated anti-mouse IgG (green). Cytokeratin staining was performed to visualize epithelial cells (red). LP, lamina propria; L, lumen; Scale bar; 50 µm.

Because only IMM013 showed a strong binding profile to small intestinal APN, this monoclonal antibody was further evaluated for its ability to serve as an antigen delivery system. Using cell lines and gut explants, the uptake of IMM03 was assessed. In contrast to an irrelevant mouse IgG1, IMM013 was clearly taken up by BHK-APN cells and by small intestinal enterocytes in the explants (**Figures 2A, B**). Some transcytosis of IMM013 occurred as can be seen by the presence of antibodies at the basolateral side of the intestinal epithelial cells.

**Figure 3:**
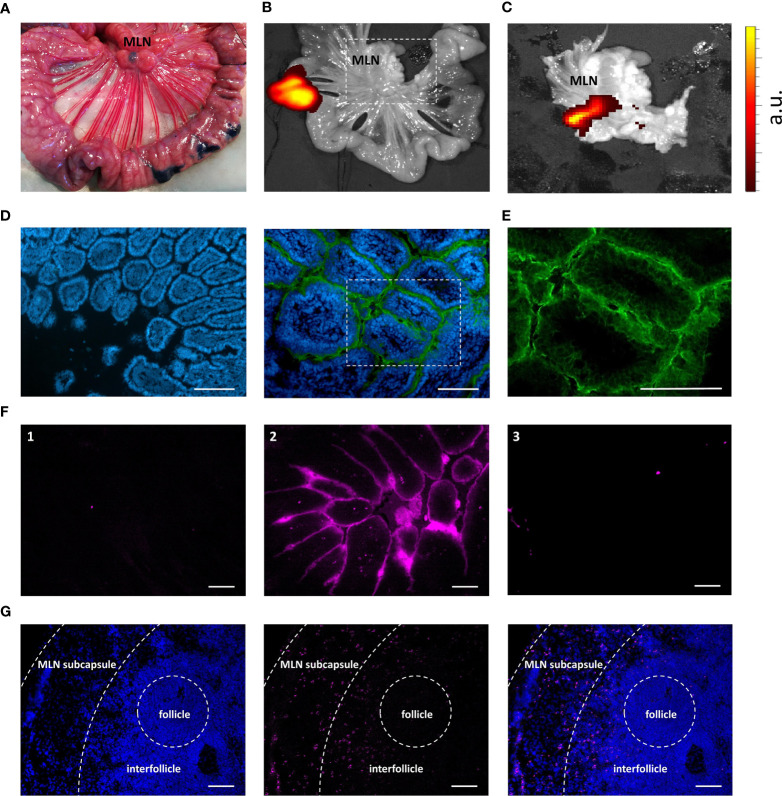
Uptake, endocytosis and migration of IMM013 from the gut mucosa to the mesenteric lymph nodes. **(A)** Ligated jejunal loop injected subserosally with Evans Blue to localize the draining mesenteric lymph nodes (MLN). Photograph taken 5 minutes after injection. **(B)** Ligated jejunal loop injected with 1 mg of IMM013-DL755. Image captured 5 hours after injection. **(C)** Squared box in b. Intensity scale from low (red) to high (yellow). a.u: arbitrary units **(D)** Immunostaining of a gut ligated loop injected with IgG1 isotype (left) and IMM013 (right); nuclei (blue), FITC-conjugated anti-mouse IgG (Green). Scale bar: 100µm. **(E)** Squared box in d. **(F)** Cryosections from ligated gut loops injected with (1) PBS, (2) IMM013-DL755 and (3) IgG1-DL755. Images taken from unprocessed cryosections. Scale bar: l00µm. **(G)** Immunohistochemistry of a draining MLN; nuclei (left, blue), IMM013-DL755 (center, magenta) and merge (right). Images taken from unprocessed cryosections. Scale bar: 100 µm. Images are representative of three animals.

**Figure 4:**
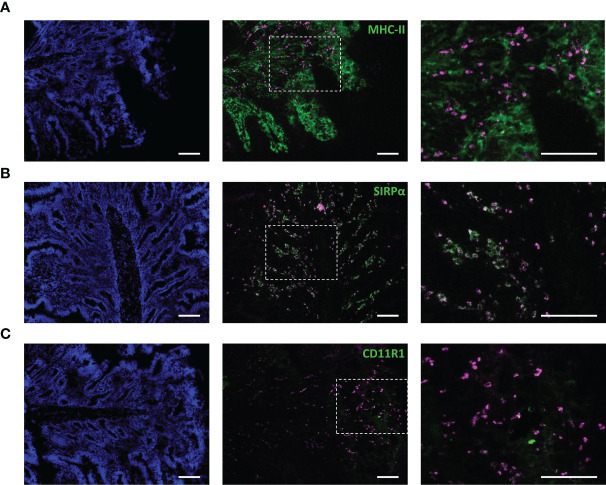
Antibody-mediated targeting to APN results in uptake by antigen presenting cells upon epithelial transcytosis. Cryosections from ligated gut loops injected with IMM013-DL755 (magenta) and incubated for 5 h. Left images showing cell nuclei (blue). Right images represent the squared boxes of the merged images. Cryosections were immunostained for **(A)** MHC-II (Green) **(B)** SIRPα (Green) and **(C)** CD11R1 (Green). Double positive cells in white. Scale bar: 100 µm. Images are representative of three animals.

To confirm the behavior of IMM013 in an *in vivo* setting, gut ligated loop experiments were performed. Since we wanted to assess if APN targeted antibodies can reach the mesenteric lymph nodes (MLN) upon epithelial transcytosis, Evans blue was injected at the edges of the gut loops to identify the draining MLN of each ligated loop (**Figure 3A**). Upon injection of DL755-labelled IMM013, its presence in the gut loop and the draining MLN was confirmed upon 5h incubation (**Figures 3B, C**). Similar to the explant results, APN targeting resulted in the endocytosis and transcytosis of the antibodies by small intestinal epithelial cells (**Figures 3D, E**). Moreover, transcytosis of IMM013 by epithelial cells resulted in the presence of mAb positive cells in the subepithelial tissue in the villi (**Figure 3F2**), implying that antigen presenting cells (APCs) phagocytosed the antibody released by the epithelial cells upon transcytosis. Furthermore, we also analyzed the distribution of these antibody-positive APCs in the draining MLN, where they were found mainly in the subcapsular and interfollicular regions (**Figure 3G**). To further investigate which cells might phagocytose the antibody upon epithelial transcytosis, tissue sections were stained with three APC markers associated with mononuclear phagocytes in the porcine gut: MHC-II, SIRP-α and CD11R1 (29). The results showed that 98% of the IMM013 positive cells expressed MHC class II, 96% expressed SIRP-α and 93% expressed CD11R1 (**Figure 4**).

### Antigen-Specific Intestinal Immune Responses After Oral Administration of APN-Targeted Antibody Constructs

To evaluate the ability of IMM013 to induce systemic and local immune responses against a linked antigen after oral delivery, several IMM013-based antibody constructs were developed. First, a fusion construct was made using the clinically relevant antigen, FedF from F18 fimbriated *E. coli* (α-APN-mIgG1-FedF). Next, the mouse IgG1 (mIgG1) Fc-domain of this construct was changed to a porcine IgA (pIgA) Fc-domain as previously described (20), in an attempt to increase antibody stability in the intestinal tract and reduce mouse IgG1-specific immune responses (α-APN-pIgA-FedF). To check for the effect of APN-targeting, a pig IgA-FedF control construct (pIgA-FedF) was also derived by rational design. Here, a single amino acid in the CDRH3 loop was mutated (G100D; MUT7), resulting in the substitution of a small non-polar amino acid into a larger polar amino acid. This single mutation completely abolished APN binding, while maintaining antibody stability. Binding and uptake characteristics of the FedF-linked fusion constructs were confirmed to be similar to IMM013 (**Supplementary Figure S1**).

**Figure 5:**
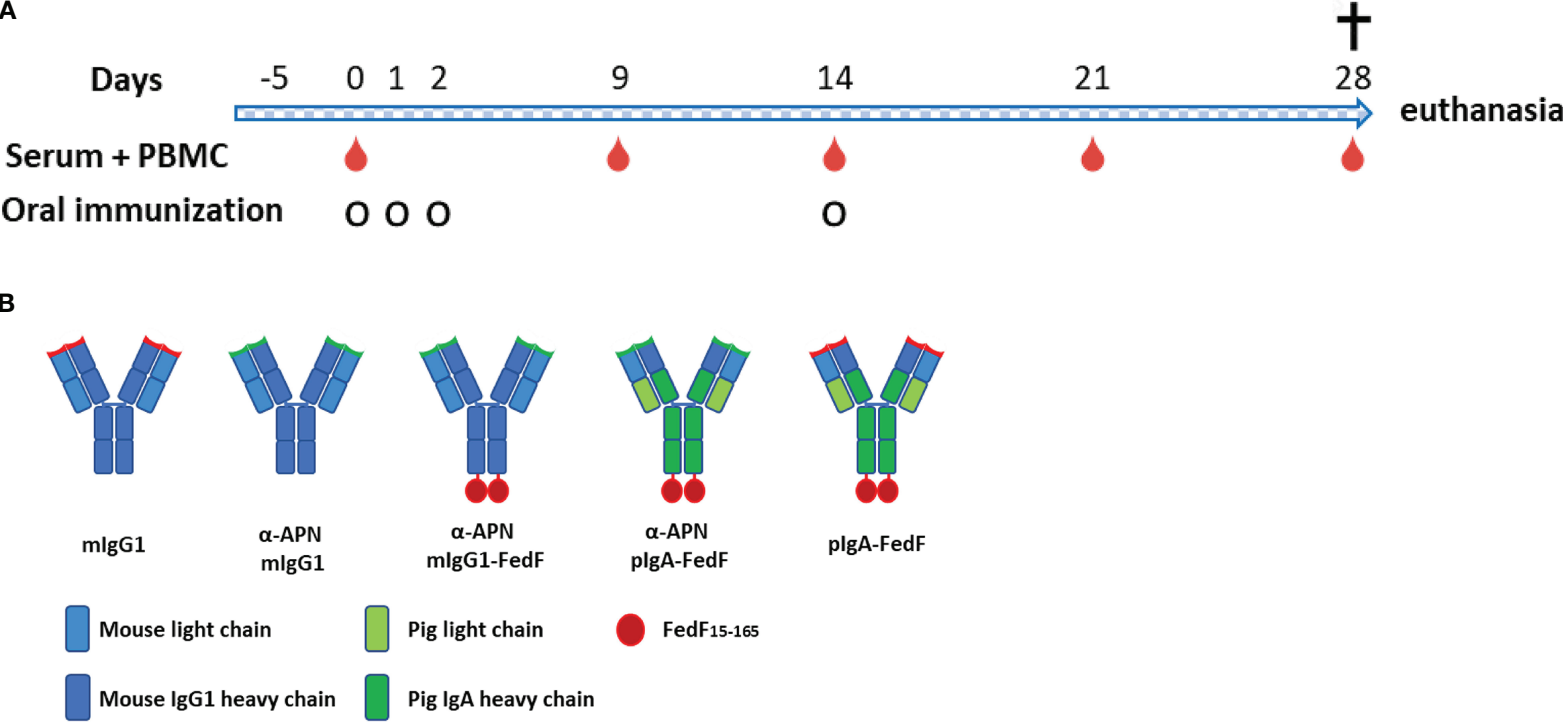
Experimental overview. **(A)** Timeline of oral immunization experiment with serum and PBMC collection days and oral immunization time points. **(B)** Overview of different antibody constructs. mIgG1, mouse IgG1; plgA, pig lgA.

**Figure 6:**
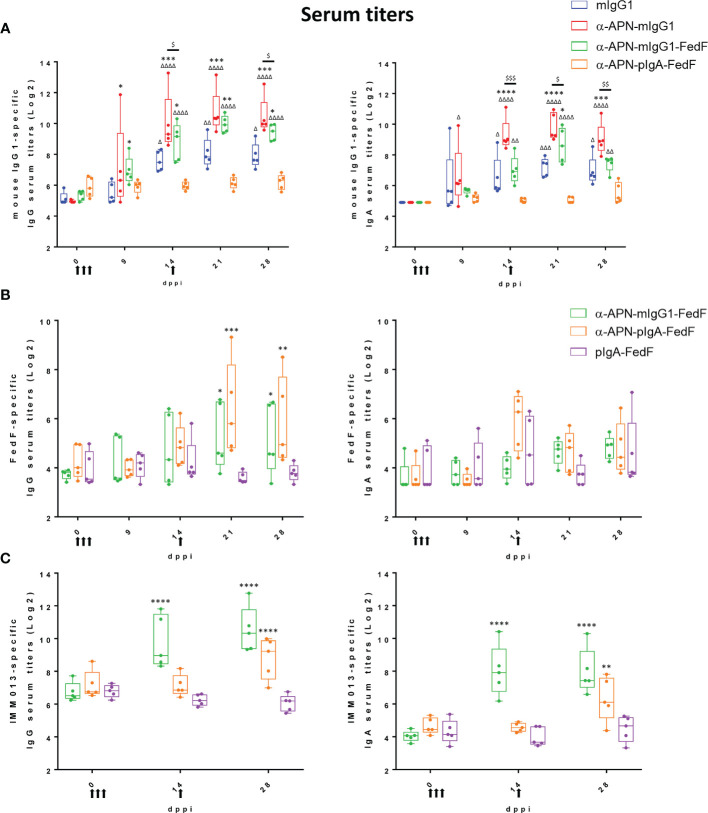
Increased serum responses after oral immunization with APN-specific antibody constructs. **(A)** mIgG1-specific, **(B)** FedF-specific and **(C)** IMM013-specific IgG (left) and IgA (right) serum titers 0, 9, 14, 21 and 28 dppi (days post primary immunization). OD: optical density. Arrows indicate days of immunization. Multiplicity adjusted p-values: ^* ,Δ,$^p < .05; **^,ΔΔ,$$^p < .01; ***^,ΔΔΔ,$$$^p < .001; ****^,ΔΔΔΔ^p < .0001; * indicates significant differences compared to **(A)** mIgG1 isotype ctrl or **(B, C)** pIgA-FedF ctrl; **(A)** Δ indicates significant differences compared to pIgA-FedF ctrl; $ indicates significant differences between α-APN-mIgG1 and α-APN-mIgG1-FedF. n=5. mIgG1, mouse IgG1; pIgA, pig IgA.

**Figure 7:**
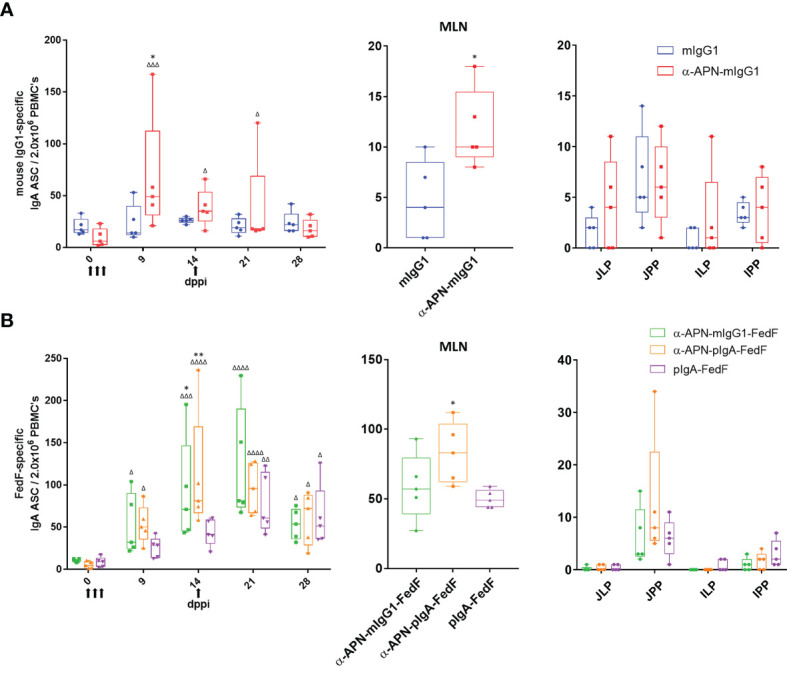
Antigen-specific antibody secreting cells after oral immunization with APN-specific antibody constructs. ELISpot of **(A)** IgG1 -and **(B)** FedF-specific IgA ASCs from PBMCs (left) isolated on 0, 9, 14, 21 and 28 dppi (days post primary immunization) and mononuclear cells isolated from mesenteric lymph nodes (middle) and intestinal tissues (right) 28 dppi. Arrows indicate days of immunization. Multiplicity adjusted p-values: *^,Δ^p < .05; **^,ΔΔ^p < .01; ^ΔΔΔ^p < .001; ^ΔΔΔΔ^p < .0001; * indicates significant differences compared to mIgG1 isotype or pIgA-FedF ctrl on same day, while Δ indicates significant differences for each group compared to day 0. n=5. mlgG1, mouse IgG1; plgA, pig IgA; MLN, mesenteric lymph nodes; JJLP, jejunal lamina propria; JJPP, Jejunal Peyer’s Patches; ILP, Ileal lamina propria; IPP, Ileal Peyer's Patches.

All constructs were subsequently used in an oral immunization experiment in weaned piglets to evaluate the effect of APN-targeting in inducing systemic and local immune responses against the antibody and the fused antigen. To this end, piglets were orally immunized with a mouse IgG1 isotype control, an APN-specific mouse IgG1 (α-APN-mIgG1), an α-APN-mIgG1-FedF fusion construct, a chimeric α-APN-pIgA-FedF fusion construct and a chimeric pIgA-FedF control antibody (**Figure 5**). The ability of these different antibody formats to elicit mouse IgG1 and FedF-specific immune responses was evaluated by ELISA and ELIspot (**Figures 6**, **7**). Here, we showed a clear increase in mouse IgG1-specific IgG and IgA serum responses at 9, 14, 21 and 28 days post primary immunization (dppi) for the APN-targeted antibodies as compared to the mIgG1 isotype control. The chimeric α-APN-pIgA-FedF fusion construct did not result in mouse IgG1-specific serum responses. Interestingly, the mouse IgG1-specific immune responses against the α-APN-mIgG1-FedF fusion construct were significantly weaker compared to the original α-APN-mIgG1, with only significant IgA serum responses observed 21 dppi. Furthermore, significantly lower mouse IgG1-specific IgG and IgA serum responses were also observed as compared to the original α-APN-mIgG1 on 14, 21 and 28 dppi (**Figure 6A**), indicating that fusion with FedF seemed to reduce mouse IgG1-specific serum responses. The targeting of FedF to APN by the antibody fusion constructs also resulted in significant FedF-specific IgG serum responses 21 and 28 dppi as compared to the pig IgA-FedF control antibody. Surprisingly, the FedF-specific IgA serum responses did not differ between groups (**Figure 6B**). Although the APN-targeted IgA-FedF construct did not result in mouse IgG1-specific immune responses, significant differences in IMM013-specific serum IgG and IgA responses could be observed compared to the pIgA-FedF control antibody, indicating that the mouse variable domain is still immunogenic and that the targeting towards APN was effective in promoting immune responses (**Figure 6C**).

To further investigate the mouse IgG1 and FedF-specific immune responses, the amount of circulating antigen-specific IgA+ antibody secreting cells (ASCs) were assessed by ELISpot (**Figure 7**). A significant increase in the number of mouse IgG1-specific IgA ASCs was found 9 dppi for the APN targeted antibody as compared to day 0 and the mouse IgG1 isotype control (**Figure 7A**). For FedF, significant increases in FedF-specific IgA ASCs as compared to day 0 were found at 9, 14, 21 and 28 dppi for the APN targeted antibody constructs, but also for the pig IgA-FedF control group at 21 and 28 dppi. Significant differences compared to the pig IgA-FedF control group could be found for the APN-targeted groups at 14 dppi (**Figure 7B**). To assess local gut immune responses, the number of antigen-specific IgA+ ASCs in small intestinal tissues were enumerated by ELISpot at 28 dppi (**Figure 7**). Here, APN targeting elicited both mIgG1- and FedF-specific IgA+ ASCs in the mesenteric lymph nodes, but not in other tissues.

### F18 Fimbriae Disrupt Antigen Presenting Capacity of Intestinal Antigen Presenting Cells

**Figure 8:**
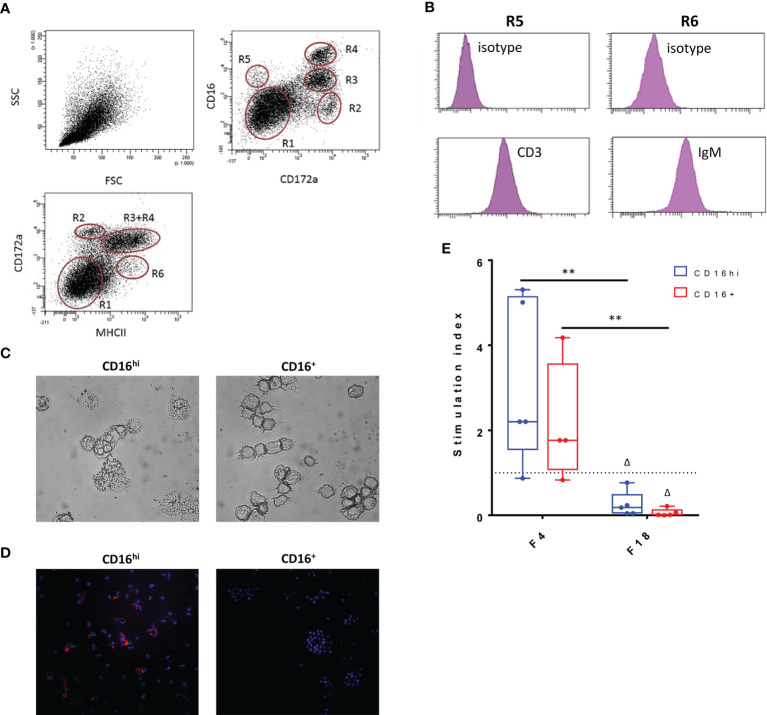
Influence of F18 fimbriae on antigen presenting capacity of intestinal antigen presenting cells. **(A)** Phenotypical analysis of small intestinal lamina propria mononuclear cells (LP MC). LP MCs were stained to determine CD172a, CD16 and MHCII expression in the live cell gate (CytoxBlue neg.) upon doublet discrimination. R1: lymphocytes; R6: MHCII^+^ activated T- and B-cells; R5: CD3^+^ T cells; R2-R4: myeloid cells. Representative plots for five separate experiments. **(B)** The LP MC population R6 consists mainly of IgM^+^ B-cells, while CD3^+^ T-cells make up the R5 population. **(C)** Morphological differences between CD16^hi^ and CD16^+^ populations. **(D)** Macrophage-specific staining (CD68; red) of CD16^hi^ and CD16^+^ population. Cell nuclei are shown in blue (Hoechst staining). **(E)** Both CD16 ^hi^ and CD16^+^ iAPCs (1.0 x 10^4^) were stimulated for 24h with the indicated agents (x-axis) and co-cultured with 1.0 x 10^5^ CD6^+^ T-cells for 5 days. Proliferative responses were measured via the incorporation of tritiated thymidin (n = 5). Control = 1432 +/- 4332 cpm, ConA = 23833 +/- 16056 cpm. Multiplicity adjusted p-values: ^Δ^p < 0.05; **p < 0.01. * indicates significant differences between the stimulated conditions (F4 and F18 fimbriae), while Δ indicates significant differences compared to the mock-stimulated iAPCs. cpm: counts per minute.

Since the data indicated that FedF might suppress immune responses, we sought to determine the cause of this immunosuppression. Given the importance of antigen-presenting cells in initiating immune responses, we hypothesized that FedF might affect the function of intestinal antigen-presenting cells (iAPC). The latter were isolated from jejunal lamina propria mononuclear cells, based on their MHCII, SIRPα and CD16 expression. Using these markers, several intestinal mononuclear cell populations could be distinguished (**Figure 8A**). Besides CD3^+^CD16^+^ intestinal T-cells (**Figure 8B**, R5) and MHCII^+^IgM^+^ B-cells (**Figure 8B**, R6), we also obtained two different antigen presenting cell populations, MHCII^+^SIRPα^+^CD16^hi^ (CD16^hi^, R3) and MHCII^+^SIRPα^+^ CD16^+^ (CD16^+^, R4). Morphological analysis revealed that in contrast to the CD16^+^ population, the CD16^hi^ cells had many vacuoles, resulting in a foamy appearance, a typical feature of macrophages (**Figure 8C**). Furthermore, the CD16^hi^ population expressed CD68, a marker specific for macrophages, while CD16^+^ cells did not (**Figure 8D**). Based on these data, we concluded that the CD16^hi^ population resembled macrophages, while the CD16^+^ population consisted of ‘bona fide’ intestinal dendritic cells. In an effort to elucidate the antigen presenting capacity of these intestinal APC populations, both the CD16^hi^ and CD16^+^ populations were stimulated with purified F4 and F18 fimbriae and cocultured with CD6^+^ T-cells. We then evaluated the T-cell proliferation-inducing ability of these stimulated cell populations. While stimulation with F4 fimbriae clearly enhanced the T-cell proliferation-inducing ability of these iAPCs, F18 fimbriae significantly inhibited the capacity of both the CD16^hi^ and CD16^+^ iAPCs to induce T-cell proliferation, as compared to mock-stimulated and F4 fimbriae-stimulated cells (**Figure 8E**). This implies that F18 fimbriae might disrupt the antigen presenting capacity of iAPCs.

## Discussion

Oral vaccination remains challenging due to the presence of the epithelial barrier and the tolerogenic responses pervading the gut immune system, which impede mounting robust immune responses to oral antigens. The targeting of vaccine antigens towards epithelial cells and antigen presenting cells might be a potential mechanism to increase the efficacy of oral vaccines by interacting with receptors that activate different signaling transduction pathways, circumventing the tolerogenic response and enhancing uptake (7, 30, 31). Our group has identified APN as an interesting target for oral antigen delivery (16–18). In this study, we evaluated the use of APN-targeted monoclonal antibodies and recombinant antibody constructs as a delivery system for vaccine antigens.

Starting from a panel of different APN-targeting mAbs, the clone IMM013 was identified as the best candidate for further *in vivo* experiments. This mAb showed the strongest binding towards the membrane-bound form of APN. Affinity measurements also showed the highest values for IMM013. Targeting APN using IMM013 resulted in endocytosis and transcytosis by intestinal epithelial cells as previously shown for APN-targeted polyclonal antibodies and single-domain nanobodies (16, 18). Upon transcytosis, the APN-targeted IMM013 mAb could be detected in subepithelial cells and in the draining mesenteric lymph nodes. Moreover, these antibody-positive subepithelial cells were also positive for MHCII, SIRP-α and CD11R1, which are present in mononuclear phagocytes. These markers, especially CD11R1, have been shown to be present on migratory cells from the lamina propria to the mesenteric lymph nodes in pigs (29, 32–34). Thus, it is tempting to speculate that upon transcytosis by epithelial cells and phagocytosis of the released antibodies by antigen presenting cells, these cells migrate to the mesenteric lymph nodes to initiate immune responses. However, cell-free transport of the released antibodies via lymph cannot be excluded.

In addition, we wanted to test the ability of antibody-mediated targeting of antigens towards intestinal APN to trigger antigen-specific immunity. Therefore, several APN-targeted recombinant antibody constructs were generated based on the IMM013 mAb and genetically linked to a clinically relevant antigen. Generated fusion constructs included an α-APN-mIgG1-FedF, a chimeric α-APN-pIgA-FedF and a chimeric pIgA-FedF not binding to APN. These constructs together with an α-APN-mIgG1 (IMM013) and a mouse IgG1 isotype control were subsequently tested in an oral vaccination experiment. As a clinically relevant antigen, the low immunogenic tip adhesin FedF of F18 fimbriated *E. coli* was chosen, as it previously failed to provoke any immune responses when orally administered to pigs (17, 19). The fusion construct was partially porcinized with an IgA Fc-tail to minimize immune responses to the antibody itself. We opted for an IgA Fc-domain for its expected higher stability in the gut environment, even in its monomeric format, as alluded to by other authors (35, 36). Both the α-APN-mIgG1 and α-APN-mIgG1-FedF fusion constructs generated strong mouse IgG1-specific serum IgG and IgA responses, with significant differences compared to the non-targeted mIgG1 isotype control antibody, indicating that targeting of the antibodies towards the epithelial membrane promoted immune responses. Interestingly, mouse IgG1-specific serum responses of the FedF-linked α-APN-mIgG1 were significantly lower compared to the original α-APN-mIgG1, especially for IgA. One possibility is that the presence of FedF decreased the immunogenicity of the carrier. This could either be due to steric hindrance of the mouse IgG1 epitopes or by a tolerogenic effect of FedF itself. To investigate this further, FACS-sorted intestinal antigen presenting cells were stimulated with purified F4 and F18 fimbriae and cocultured with CD6^+^ T-cells. As expected, T-cell proliferation was stimulated after induction with F4 fimbriae. However, after induction with F18 fimbriae, T-cell proliferation was drastically reduced, implying that these F18 fimbriae might disrupt the antigen presenting capacity of intestinal antigen presenting cells. The exact mechanism behind this process however remains unknown. Fucosylated glycosphingolipids might play a role as FedF interacts with these molecules, but this should be further investigated (37).

We provide evidence that targeting of FedF towards intestinal APN also increased FedF-specific immune responses. Significant differences in FedF-specific IgG serum responses, but not IgA serum responses could be observed for the APN-targeted FedF fusion constructs as compared to the non-targeted pIgA-FedF. Interestingly, mouse IgG1- and IMM013-specific IgG serum responses were already observed 9 or 14 dppi, while significant increases in FedF-specific serum responses were only observed after the boost at 21 and 28 dppi. These data indicate that FedF itself is not a good immunogen and that a booster immunization is required to observe significant responses. Despite the lack of IgA serum responses, significant increases in the number of IgA ASCs in the PBMCs and MLNs were found as compared to the control groups. This discrepancy in IgG1- and FedF-specific immune responses is remarkable and again points to the ability of FedF to modulate immune responses.

Although no mouse IgG1-specific immune responses could be observed for the porcine IgA-FedF constructs, we could still detect significant IMM013-specific IgG and IgA serum responses for the α-APN-pIgA-FedF construct, compared to its pIgA-FedF control. These data indicate that the mouse variable domain is still immunogenic and that the targeting towards APN was effective in promoting immune responses. Although no FedF-specific IgA serum responses could be observed, we did observe significant IMM013-specific IgA serum responses, again indicating that FedF has immunosuppressive effects and decreases the immunogenicity of the fusion construct.

Another interesting observation is the ability of the chimeric α-APN-pIgA-FedF construct to elicit stronger FedF-specific IgA immune responses as compared to the α-APN-mIgG1-FedF fusion construct. Although monomeric IgA does not provide the same protection as SIgA against the harsh intestinal environment, some studies have shown that monomeric IgA is more stable than IgG (35, 36, 38). Differences in stability between the fusion constructs could explain the observed variation in immune responses, since cleavage into Fab and Fc fragments would prevent the targeting of the linked antigen towards APN. Although a proton-pump inhibitor was administered to minimize degradation of the antibody constructs, some degradation might still occur. Antibody stability in the gut could be further enhanced by adding inhibitors of digestive enzymes, engineering antibodies to be more resistant to proteolytic cleavage or encapsulating them to provide further protection (39).

This study showed that immunization with APN-targeted mouse IgG1-FedF and pig IgA-FedF antibodies increased FedF-specific IgG serum levels and that ASCs isolated from the draining mesenteric lymph nodes were able to secrete FedF-specific IgA. Although FedF on its own is not immunogenic when given orally, FedF-conjugates with MBP or F4-fimbriae did provide some protection against infection (19, 40). In these studies however, no increase in FedF-specific serum titers were observed. In the current study, no challenge experiment was performed to assess protection, but both serum IgG titers and gut-derived IgA ASCs were increased and these correlate with protection against challenge infection (41).

In conclusion, we observed that F18 fimbriae can disrupt the antigen presenting capacity of small intestinal antigen presenting cells and that the antibody-mediated selective delivery of the F18 fimbrial tip adhesin FedF, resulted in FedF-specific systemic and local immune responses. Our results confirm that targeting of antigens towards the intestinal membrane receptor APN can promote both systemic and mucosal immune responses upon oral administration. We showed that targeting of APN promotes uptake by the epithelial barrier and that this provides a promising platform for the delivery of biologicals towards the gut tissues and beyond.

## Data Availability Statement

The raw data supporting the conclusions of this article will be made available by the authors, without undue reservation.

## Ethics Statement

The animal study was reviewed and approved by Ethical Committee of the Faculties of Veterinary Medicine and Bioscience Engineering of Ghent University (EC 2017-121, 2018-04 and 2018-61).

## Author Contributions

EC and BD conceived the idea and designed the research. HV performed the cloning, production, purification and characterization experiments. RS performed the gut-ligated loop experiments. NS provided help for imaging using the IVIS Lumina II system. HV and RS performed the immunohistochemical analysis, the *in vivo* experiment and data analysis. HV and RS wrote the manuscript with contributions from EC and BD. All authors reviewed the manuscript before submission. All authors contributed to the article and approved the submitted version.

## Funding

HV and RS are recipients of a fellowship from the Ghent University (BOF15/GOA/031). BD was supported by a postdoctoral grant of F.W.O-Vlaanderen.

## Conflict of Interest

The authors declare that the research was conducted in the absence of any commercial or financial relationships that could be construed as a potential conflict of interest.

## Publisher’s Note

All claims expressed in this article are solely those of the authors and do not necessarily represent those of their affiliated organizations, or those of the publisher, the editors and the reviewers. Any product that may be evaluated in this article, or claim that may be made by its manufacturer, is not guaranteed or endorsed by the publisher.
